# Supplementary material for: A mitochondrial surveillance mechanism activated by SRSF2 mutations in hematologic malignancies
Source: J Clin Invest. 2024 May 7;134(12):e175619. doi: 10.1172/JCI175619 (PMC11178535; doi:10.1172/JCI175619)
Supplement: Unedited blot and gel images [file jci-134-175619-s087.pdf]

Full unedited gel for Fig 4e (agarose gel)

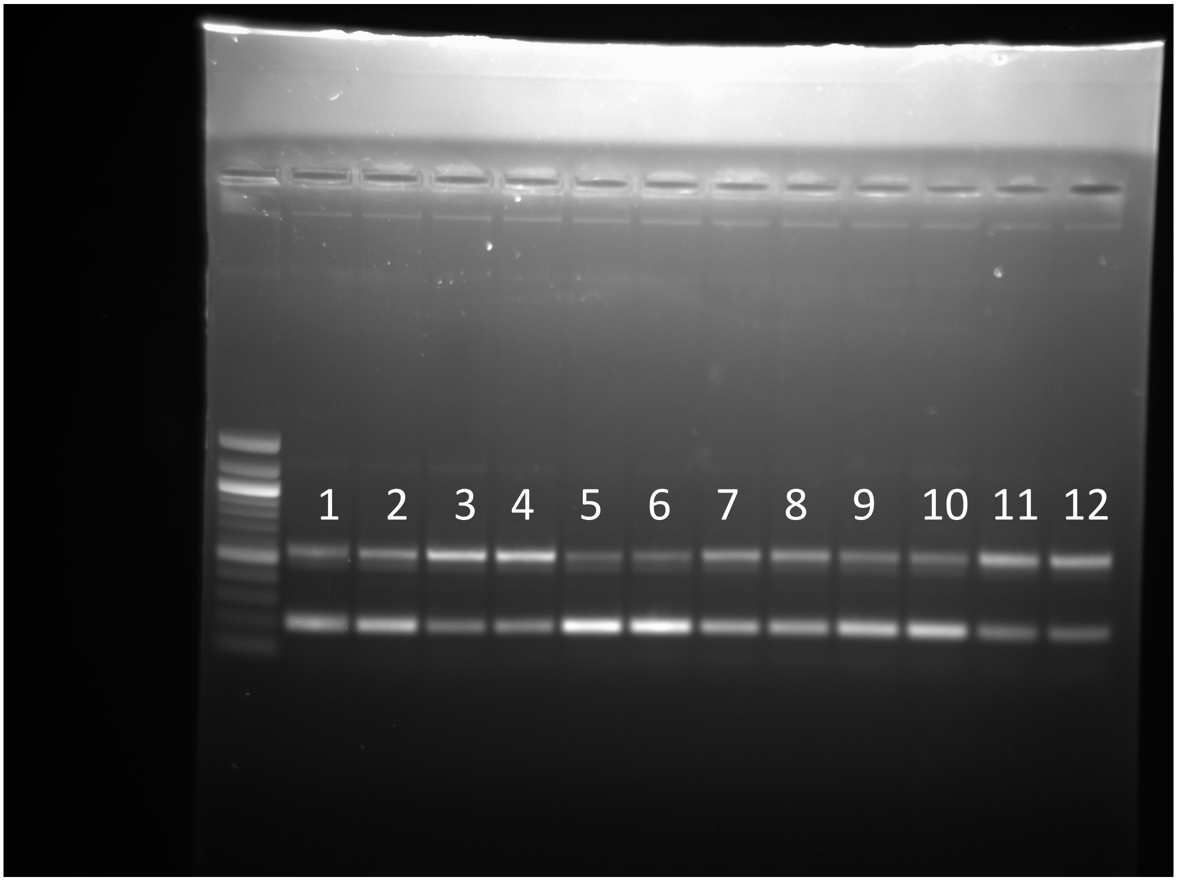

Lane 1: WT, DMSO
Lane 2: WT, DMSO
Lane 3: WT, CHIR
Lane 4: WT, CHIR
Lane 5: SRSF2$^{P95H/+}$, DMSO
Lane 6: SRSF2$^{P95H/+}$, DMSO
Lane 7: SRSF2$^{P95H/+}$, CHIR
Lane 8: SRSF2$^{P95H/+}$, CHIR
Lane 9: SF3B1$^{K700E/+}$, DMSO
Lane 10: SF3B1$^{K700E/+}$, DMSO
Lane 11: SF3B1$^{K700E/+}$, CHIR
Lane 12: SF3B1$^{K700E/+}$, CHIR

Full unedited gel for Fig 4f (agarose gel)

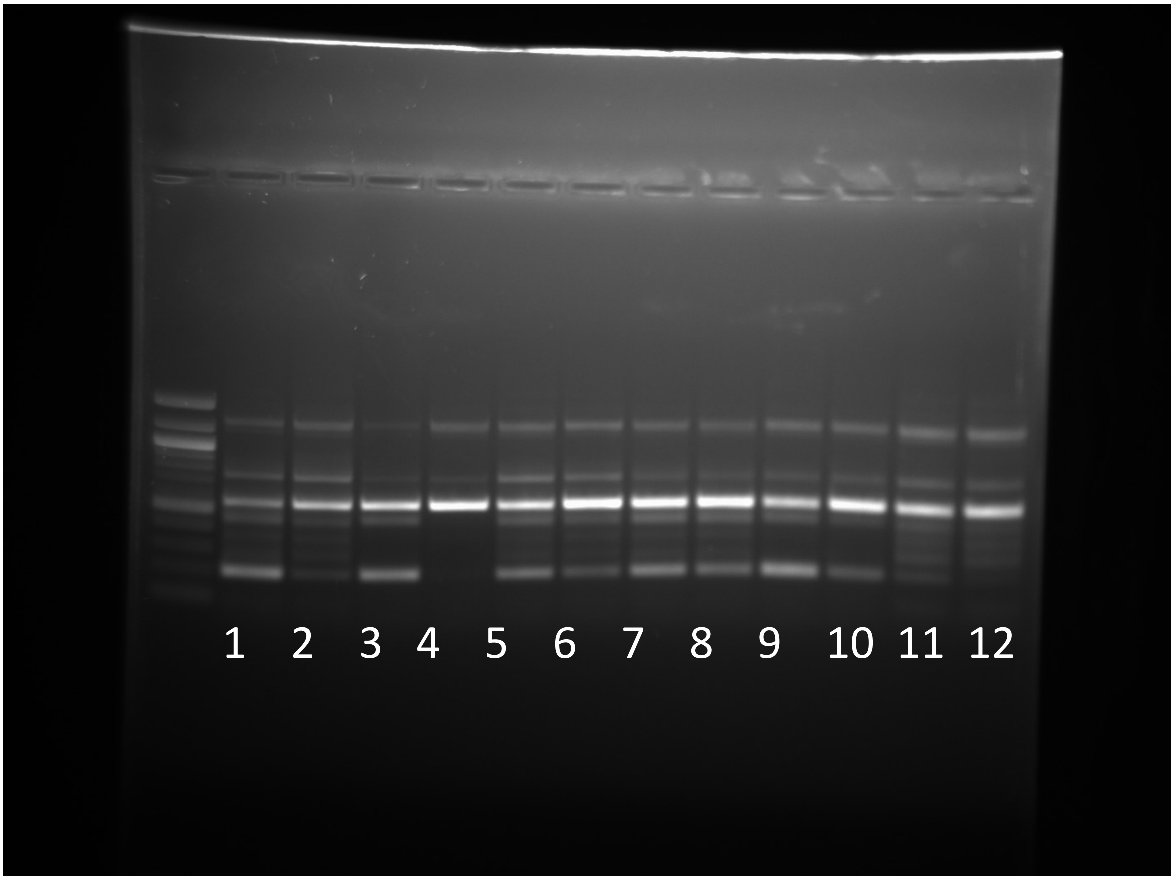

Lane 1: Normal CD34+, patient#1, DMSO
Lane 2: Normal CD34+, patient#1, CHIR
Lane 3: Normal CD34+, patient#2, DMSO
Lane 4: Normal CD34+, patient#2, CHIR
Lane 5: AML, patient#1, DMSO
Lane 6: AML, patient#1, CHIR
Lane 7: AML, patient#2, DMSO
Lane 8: AML, patient#2, CHIR
Lane 9: CMML, patient#1, DMSO
Lane 10: CMML, patient#1, CHIR
Lane 11: CMML, patient#2, DMSO
Lane 12: CMML, patient#2, CHIR

Full unedited gel for Fig 4g (agarose gel)

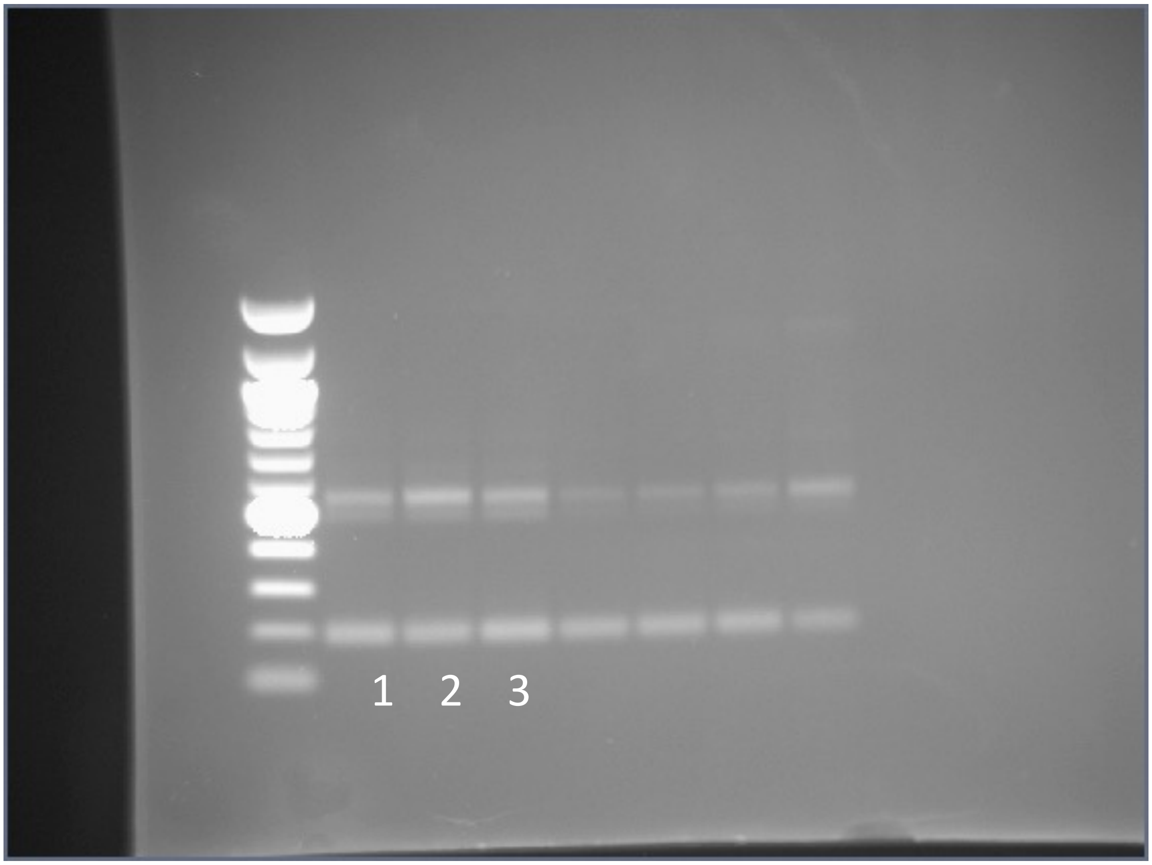

Lane 1: WT
Lane 2: DKO
Lane 3: DKO+GSK-3β

Full unedited gel for Fig 4h (agarose gel)

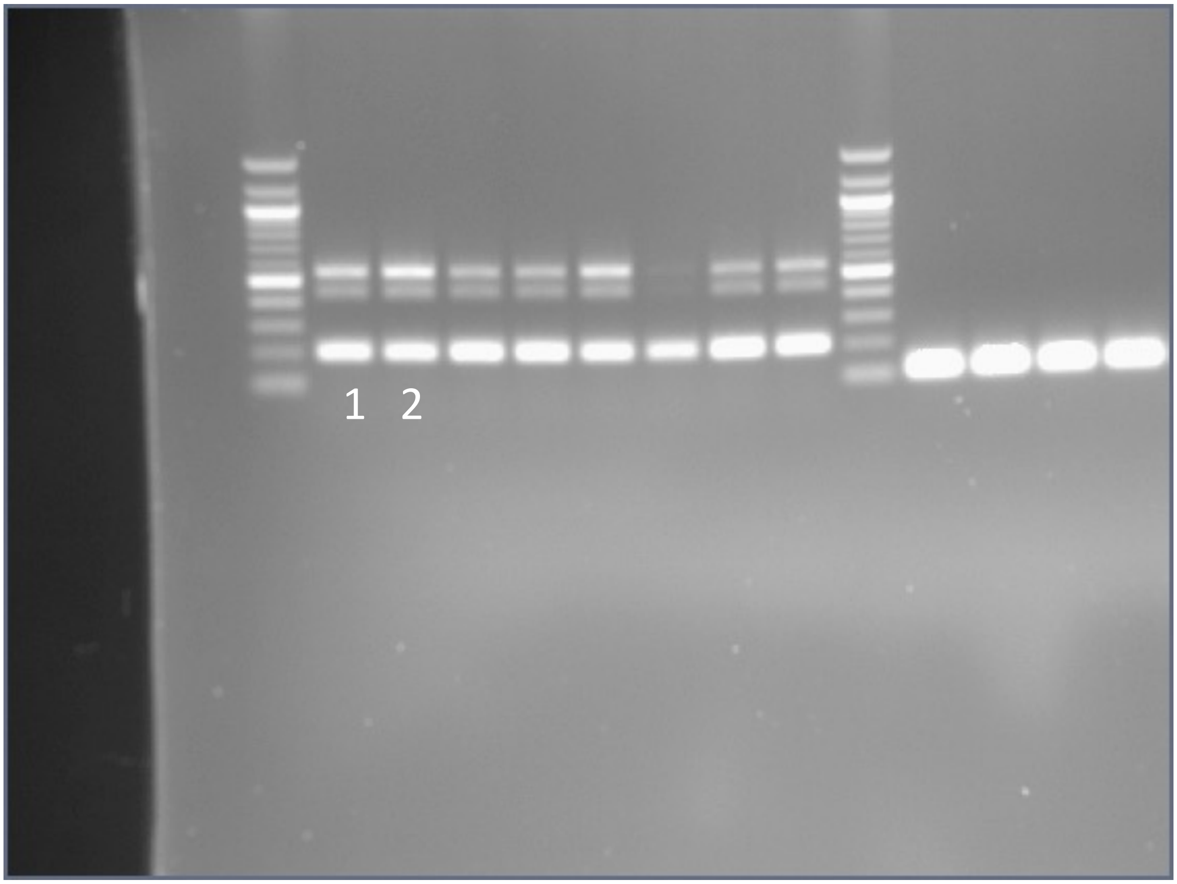

Lane 1: shNT
Lane 2: shUPF1

Full unedited gel for Figure 5e (Western blot)

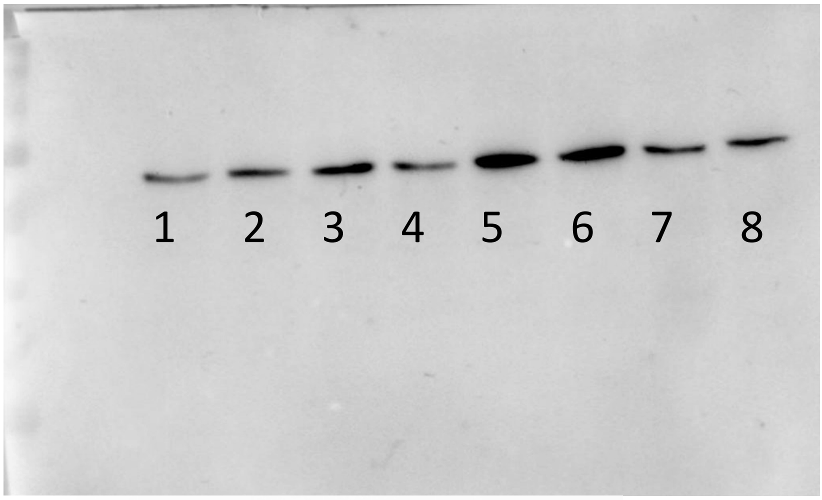

PINK1 (Invitrogen, PA5-86941)

Lane 1: WT
Lane 2: WT, CHIR, 2h
Lane 3: WT, CHIR, 8h
Lane 4: WT, CHIR, 24h
Lane 5: $SRSF2^{P95H/+}$
Lane 6: $SRSF2^{P95H/+}$, CHIR, 2h
Lane 7: $SRSF2^{P95H/+}$, CHIR, 8h
Lane 8: $SRSF2^{P95H/+}$, CHIR, 24h

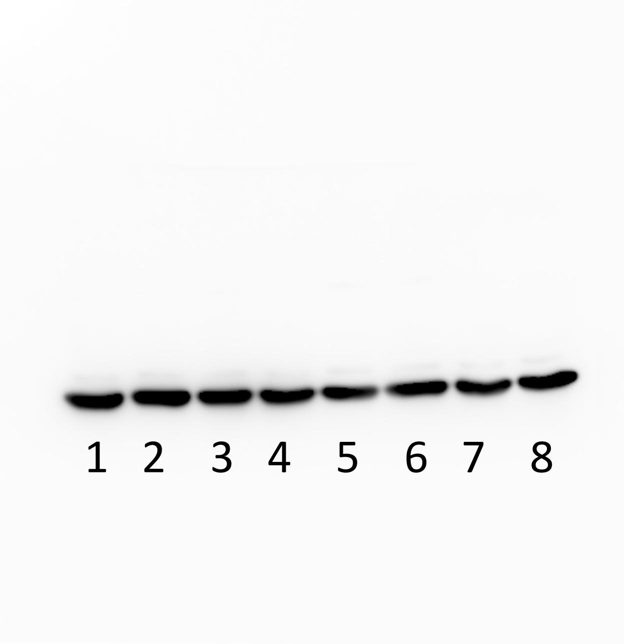

GAPDH (Ab: CST #2118)

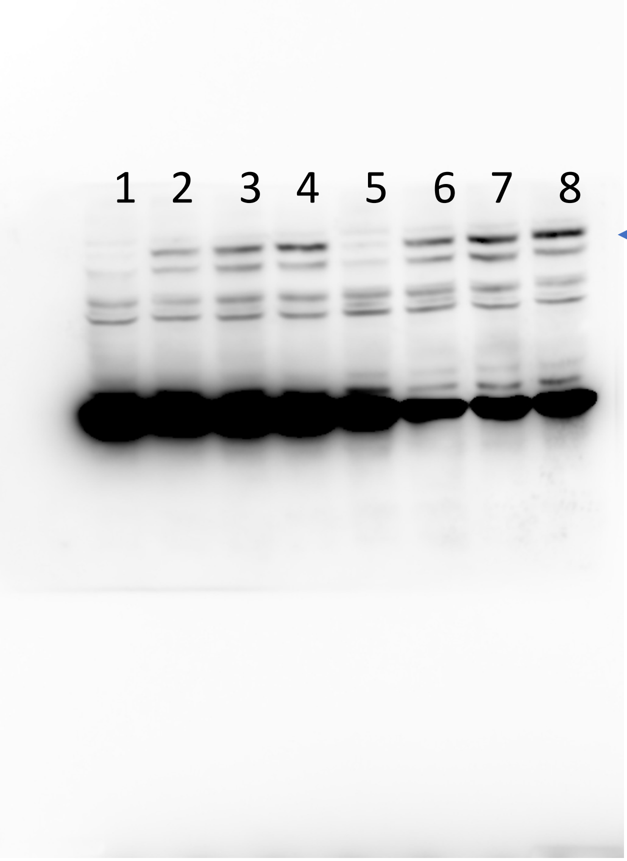

β-Catenin (Ab: CST #9562)

Full unedited gel for Fig 6k (agarose gel)

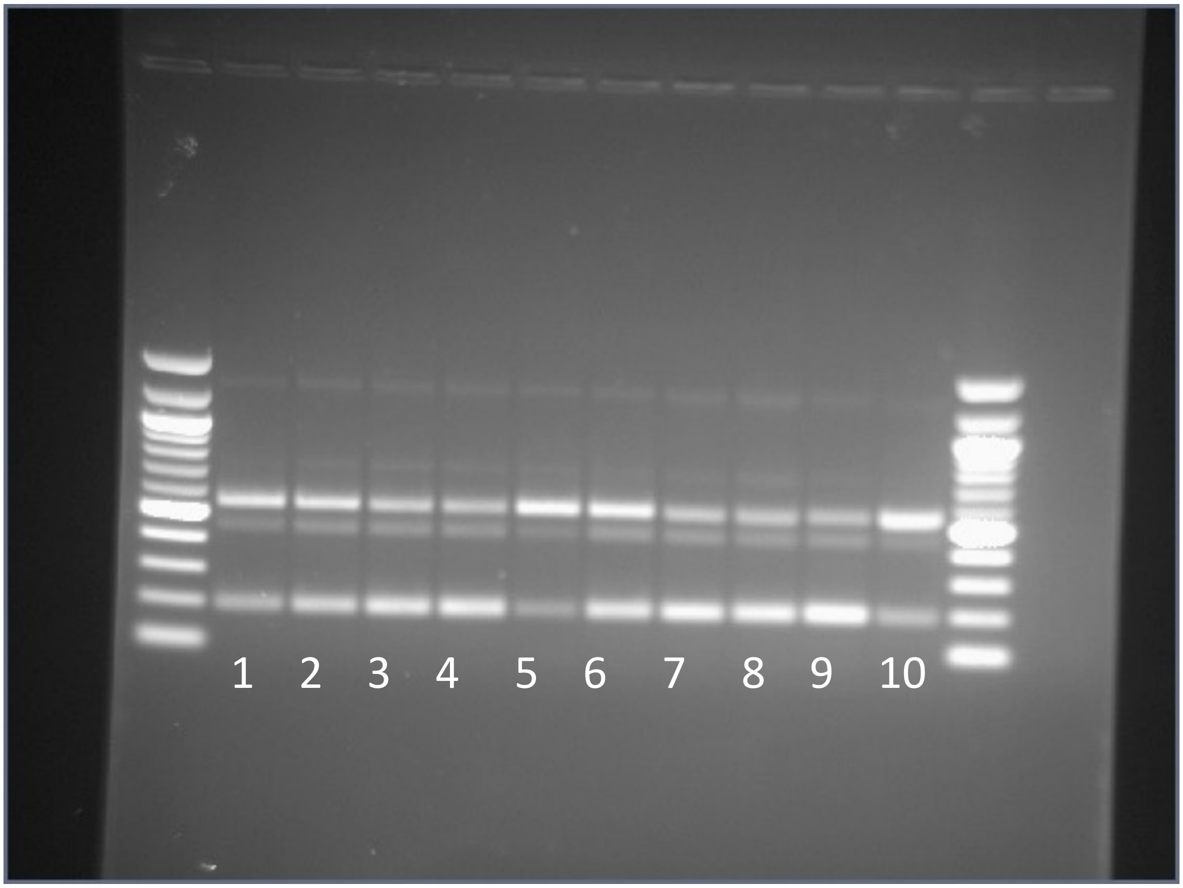

Lane 1: WT, DMSO
Lane 2: WT, CCCP 2uM
Lane 3: WT, CCCP 5uM
Lane 4: WT, CCCP 10uM
Lane 5: WT, CHIR
Lane 6: $SRSF2^{P95H/+}$, DMSO
Lane 7: $SRSF2^{P95H/+}$, CCCP 2uM
Lane 8: $SRSF2^{P95H/+}$, CCCP 5uM
Lane 9: $SRSF2^{P95H/+}$, CCCP 10uM
Lane 10: $SRSF2^{P95H/+}$, CHIR

Full unedited gel for Figure 7d (Western blot)

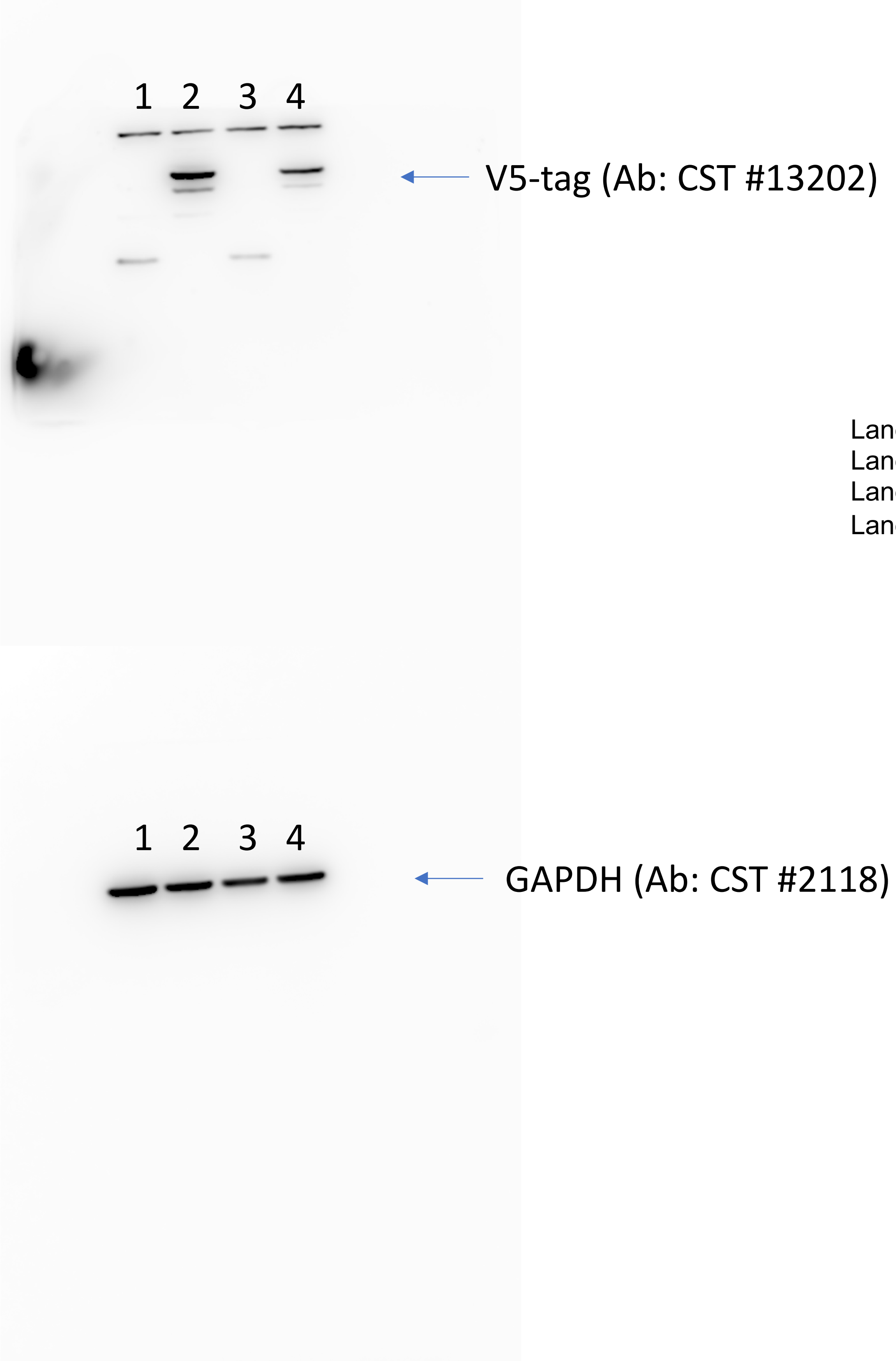

Lane 1: WT, Vector
Lane 2: WT, PINK1-OE
Lane 3: SRSF2$^{P95H/+}$, Vector
Lane 4: SRSF2$^{P95H/+}$, PINK1-OE

Full unedited gel for S Fig 1e (Western blot)

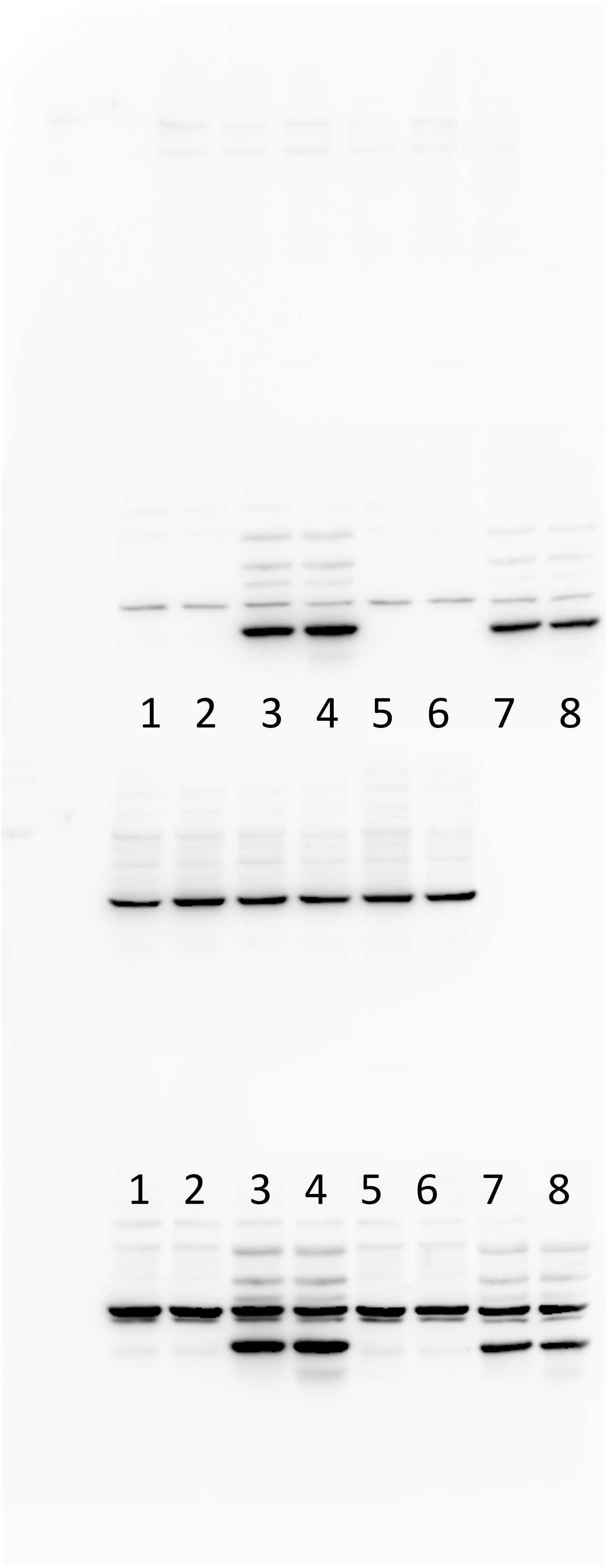

Lane 1: WT, mCherry-, DMSO
Lane 2: WT, mCherry-, CHIR
Lane 3: WT, mCherry+, DMSO
Lane 4: WT, mCherry+, CHIR
Lane 5: SRSF2$^{P95H/+}$, mCherry-, DMSO
Lane 6: SRSF2$^{P95H/+}$, mCherry-, CHIR
Lane 7: SRSF2$^{P95H/+}$, mCherry+, DMSO
Lane 8: SRSF2$^{P95H/+}$, mCherry+, CHIR

← Flag-tag (Ab: CST#14793)

← β-actin (Ab: Sigma, A5441)

Full unedited gel for S Fig 3a (Western blot)

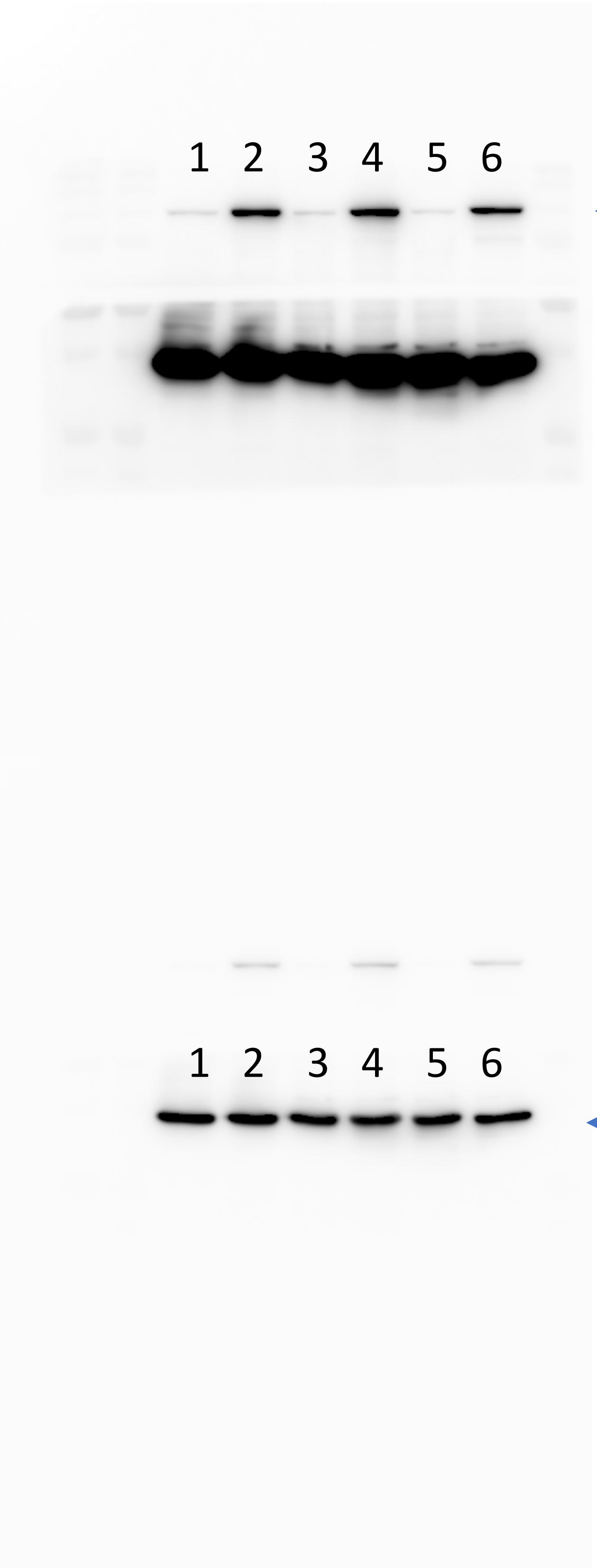

β-Catenin (Ab: CST #9562)

GAPDH (Ab: CST #2118)

Lane 1: WT, DMSO
Lane 2: WT, CHIR
Lane 3: SRSF2$^{P95H/+}$, DMSO
Lane 4: SRSF2$^{P95H/+}$, CHIR
Lane 5: SF3B1$^{K700E/+}$, DMSO
Lane 6: SF3B1$^{K700E/+}$, CHIR

Full unedited gel for S Fig 3a (agarose gel)

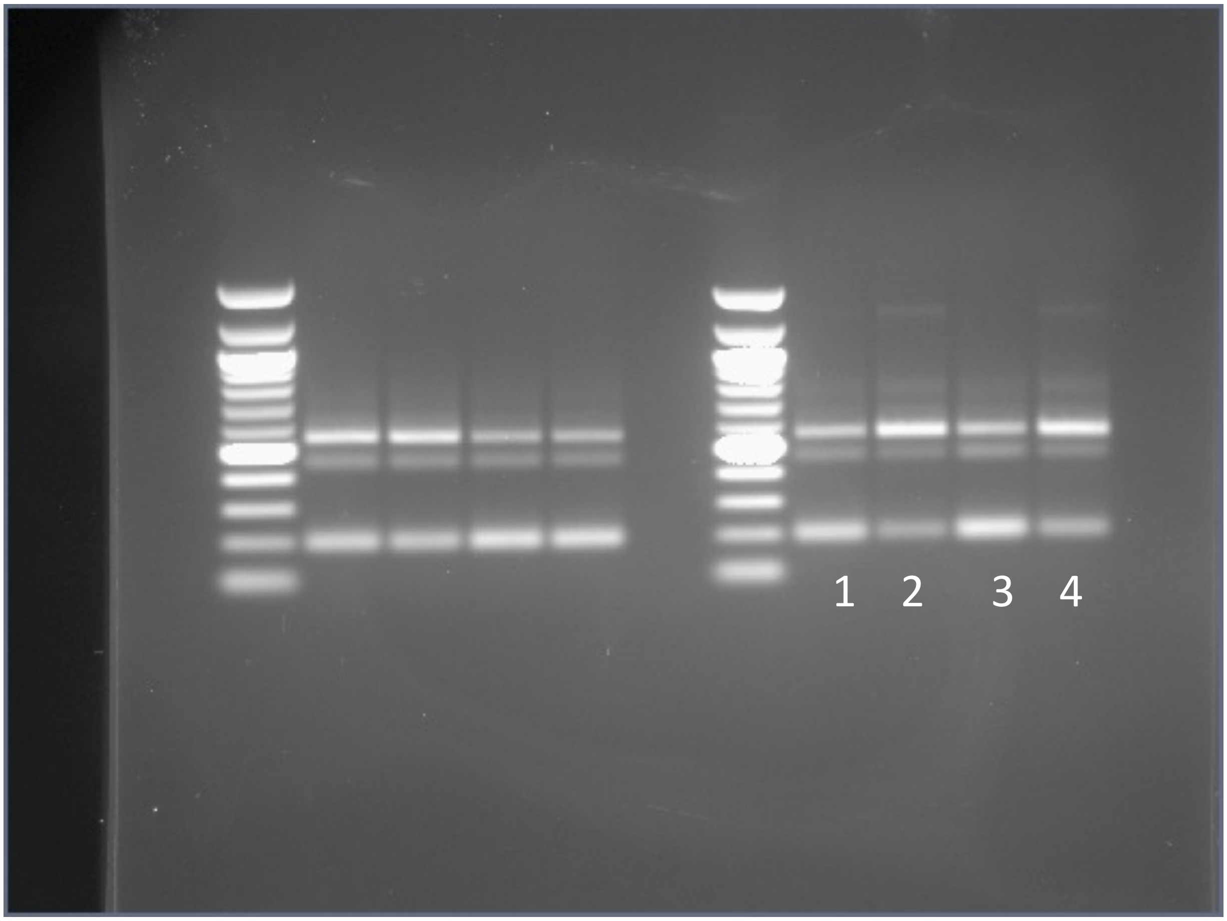

Lane 1: OE-SRSF2^{WT}, DMSO
Lane 2: OE-SRSF2^{WT}, CHIR
Lane 3: OE-SRSF2^{P95H}, DMSO
Lane 4: OE-SRSF2^{P95H}, CHIR

Full unedited gel for S Fig 4 (agarose gel)

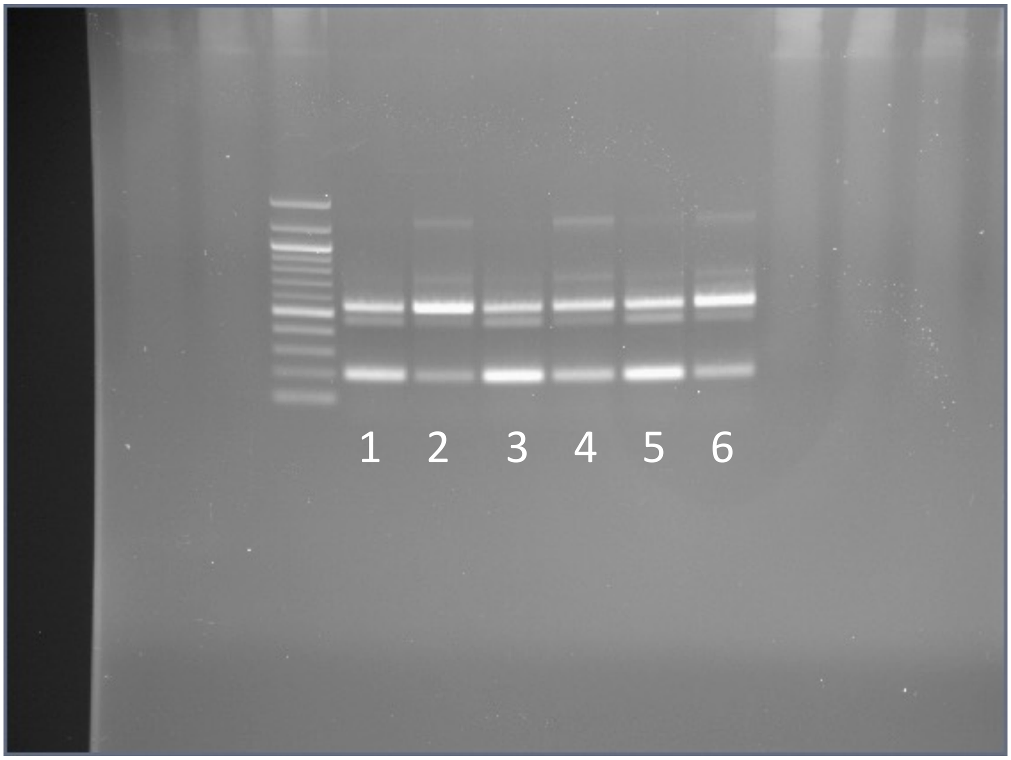

Lane 1: WT, DMSO
Lane 2: WT, AR-A014418
Lane 3: SRSF2$^{P95H/+}$, DMSO
Lane 4: SRSF2$^{P95H/+}$, AR-A014418
Lane 5: SF3B1$^{K700E/+}$, DMSO
Lane 6: SF3B1$^{K700E/+}$, AR-A014418

Full unedited gel for S Fig 5a (agarose gel)

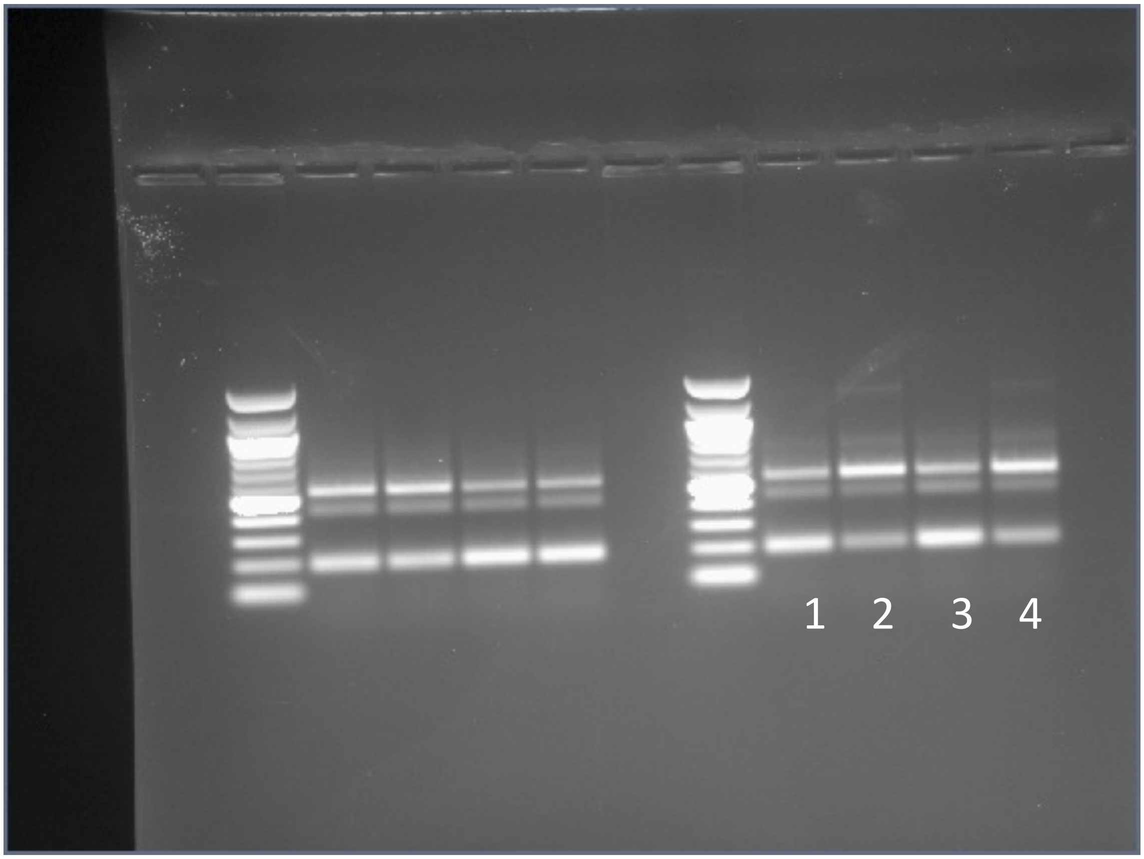

Lane 1: WT-SRSF2 OE, DMSO
Lane 2: WT-SRSF2 OE, CHIR
Lane 3: P95H-SRSF2 OE, DMSO
Lane 4: P95H-SRSF2 OE, CHIR
